# Dynamics of Deep Water and N Uptake of Oilseed Rape (*Brassica napus* L.) Under Varied N and Water Supply

**DOI:** 10.3389/fpls.2022.866288

**Published:** 2022-04-29

**Authors:** Guanying Chen, Camilla Ruø Rasmussen, Dorte Bodin Dresbøll, Abraham George Smith, Kristian Thorup-Kristensen

**Affiliations:** ^1^Department of Plant and Environmental Sciences, University of Copenhagen, Copenhagen, Denmark; ^2^Earth and Life Institute, Université Catholique de Louvain, Ottignies-Louvain-la-Neuve, Belgium; ^3^Department of Computer Science, University of Copenhagen, Copenhagen, Denmark

**Keywords:** *Brassica napus*, deep root, nitrogen use efficiency, water uptake, dual-labeling

## Abstract

Enhanced nitrogen (N) and water uptake from deep soil layers may increase resource use efficiency while maintaining yield under stressed conditions. Winter oilseed rape (*Brassica napus* L.) can develop deep roots and access deep-stored resources such as N and water to sustain its growth and productivity. Less is known of the performance of deep roots under varying water and N availability. In this study, we aimed to evaluate the effects of reduced N and water supply on deep N and water uptake for oilseed rape. Oilseed rape plants grown in outdoor rhizotrons were supplied with 240 and 80 kg N ha^−1^, respectively, in 2019 whereas a well-watered and a water-deficit treatment were established in 2020. To track deep water and N uptake, a mixture of ^2^H_2_O and Ca(^15^NO_3_)_2_ was injected into the soil column at 0.5- and 1.7-m depths. δ^2^H in transpiration water and δ^15^N in leaves were measured after injection. δ^15^N values in biomass samples were also measured. Differences in N or water supply had less effect on root growth. The low N treatment reduced water uptake throughout the soil profile and altered water uptake distribution. The low N supply doubled the ^15^N uptake efficiency at both 0.5 and 1.7 m. Similarly, water deficit in the upper soil layers led to compensatory deep water uptake. Our findings highlight the increasing importance of deep roots for water uptake, which is essential for maintaining an adequate water supply in the late growing stage. Our results further indicate the benefit of reducing N supply for mitigating N leaching and altering water uptake from deep soil layers, yet at a potential cost of biomass reduction.

## Introduction

Nitrogen (N) and water are the main factors that can be modified in agricultural production and have been widely documented for their crucial roles in determining yield (Mueller et al., 2012; Sinclair and Rufty, 2012). Inadequate supply of N and water leads to yield loss, whereas the loss of excess N from agricultural land also causes environmental problems. Therefore, improving our understanding of water and N use is crucial for sustainable agricultural production.

Deep-rooted crops have shown great potential in enhancing N and water uptake, as well as improving yield (Wasson et al., 2012; Thorup-Kristensen et al., 2020a). In arable cropping systems, growing crops with deep roots as catch crops after the main crop is a useful way to reduce N leaching for their beneficial effects on taking up and retaining the excess N left in the soil (Thorup-Kristensen et al., 2003). However, deep root development and function are highly sensitive to the environment (Lynch and Wojciechowski, 2015; Griffiths et al., 2022). Management and environmental factors such as N and water supply affect plant growth, soil N, and water availability and subsequently affect root uptake.

Optimum N supply improves overall plant growth and yield (Asare and Scarisbrick, 1995; Khan et al., 2017), but the advantages of N supply on root growth and N uptake seem ambiguous. Svoboda and Haberle (2006) observed no effect of N fertilization on root length and root length distribution of winter wheat (*Triticum aestivum* L.) in soil layers. However, when part of the roots were exposed to N-rich patches, root proliferation was observed (Drew et al., 1973). Higher concentrations of supplied N have also resulted in morphologic changes such as the extension of main roots and growth of laterals (Drew et al., 1973). These changes led to increased root length and weight densities in the patch, resulting in more N capture from the patch (Hodge et al., 1999). Plant root growth and total N uptake may be enhanced by a higher fertilization rate (Lynch et al., 2012), whereas N-deficient plants may utilize the supplied N more efficiently. It has been reported that nitrogen uptake efficiency in oilseed rape (*Brassica napus* L.) and winter wheat can decline with increasing N fertilizer rate (Rathke et al., 2006; Rasmussen et al., 2015). Riar et al. (2020) found that, independent of irrigation, oilseed rape plants fertilized with 100 kg N ha^−1^ had higher N uptake efficiency than those fertilized with 200 kg N ha^−1^. To achieve maximum yields, usually more than 200 kg N ha^−1^ N fertilizer is given to oilseed rape (Sieling and Kage, 2010). However, the higher application of N fertilizer is often accompanied by environmental concerns. Therefore, maximizing the N uptake efficiency is highly desirable.

Nitrogen supply is important for improving water use. Increased N application has been found to increase the water consumption of wheat and oilseed rape (Taylor et al., 1991; Waraich et al., 2011). Compared with no or less fertilization, adequately fertilized crops usually grow more vigorously and have larger leaf areas, increasing transpiration and decreasing soil evaporation. In the meantime, photosynthetic carbon assimilation is significantly increased by N application, leading to higher biomass production. Further, as Li et al. (2009) pointed out, N application enhances crop water use efficiency due to the increase in biomass production per mm water use.

Water availability strongly affects crop growth, especially canopy development, but it also influences root growth. Bloom et al. (1985) hypothesized that plant root growth may be stimulated under drought to enhance or maintain the capacity for acquiring water. Accordingly, Vandoorne et al. (2012) found that although the total root length of chicory (*Cichorium intybus* L.) decreased under water-deficit conditions, the overall root profile developed deeper, and root water uptake from wetter and deeper horizons compensated for low uptake in the shallow soil. Simulations conducted by Landl et al. (2019), also confirmed that increasing rooting depth benefited total root water uptake in the dry season, leading to lower transpiration deficits. Furthermore, Álvarez et al. (2011) and Li et al. (2011) observed a significant increase in water use efficiency during water deficit.

In addition to altering the root growth and water acquisition, water status affects plant N uptake in various ways. Under the same fertilization rate, Riar et al. (2020) showed that irrigation management enhanced subsoil moisture and N mobility, which further improved oilseed rape's N uptake and use efficiency by 40%. Water deficit also affects nitrogen demand, nitrogen availability, and nitrogen assimilation and partitioning of the assimilates (Sadras et al., 2016). The reduced shoot growth driven by water deficit reduces plant nitrogen demand and tends to decrease nitrogen use efficiency if N inputs are not reduced correspondingly (Quemada and Gabriel, 2016). The availability and supply of soil N can be limited by soil dryness due to reduced soil organic N mineralization (Jensen et al., 1997) and restricted nitrate movement by both mass flow and diffusion (Plett et al., 2020). Water deficit could also diminish nitrate reductase activity, hence reducing plant N assimilation (Gonzalez-Dugo et al., 2010). Moreover, assimilates tend to translocate to the roots rather than the shoots in the case of water deficiency (Li et al., 2011). In summary, both N and water supply can affect root growth, water, and N use.

Deep roots are not assumed to be as important as shallow roots for N and water uptake, as the roots reach the subsoil layers late in the growing period and are not able to develop as high densities there as in the topsoil. Existing studies show that resource deficiencies in topsoil may lead to increased resource uptake from subsoil, indicating the uptake potential of deep roots under stressed conditions (Kuhlmann et al., 1989; Haberle et al., 2006; Kirkegaard et al., 2007). However, it is less clear to what extent deep root growth, deep uptake of N, and water, as well as their uptake dynamics, are affected by the varied N and water availability for a crop. Studies of deep root growth, water, and N use under different N and water regimes could increase our understanding of the functions of deep roots. In addition, such studies would increase the understanding of the contribution of deep roots to crop N and water supply and to reduce N leaching losses, and how this is affected by crop management.

Oilseed rape is known for its high capacity for N, water uptake, and potential to develop roots in soil layers below 2 m (Dresbøll et al., 2016; Kirkegaard et al., 2021). In this study, we used oilseed rape as the model crop and examined how N and water supply affect the root growth, utilization of N and water from deep soil layers, and N and water uptake dynamics in the subsoil. It was hypothesized that (I) N and water deficiency in topsoil stimulate root growth in deeper soil layers; (II) lower N availability in the upper soil layer reduces total water uptake, but enhances N uptake from the subsoil; (III) lower water availability in the upper soil layer reduces the N uptake from the whole soil profile, but increases water uptake from the subsoil.

## Materials and Methods

### Experimental Facility

A number of two consecutive experiments were conducted in the seasons 2018–2019 and 2019–2020 using the rhizobox facility (Thorup-Kristensen et al., 2020b) at the University of Copenhagen in Taastrup, Zealand, Denmark (55°40′ N; 12°18′ E). The facility consists of rhizoboxes that allow observations of root growth and root activity down to 4-m depth. The growth medium was field soil. In both years, the topsoil was replaced right before planting (Table 1). The rhizoboxes are rectangular columns of 1.2 × 0.6 m, divided into an east- and a west-facing chamber, each with a surface area of 1.2 × 0.3 m. The front of the chambers is divided into 20 panels by metal frames covered by removable white foamed PVC boards, allowing root observations through transparent acrylic boards. The acrylic boards can be removed for sampling and measurements that require direct soil contact. For further details on the facility, refer to Rasmussen et al. (2020) and Thorup-Kristensen et al. (2020b).

**Table 1:** Characteristics of the soil in rhizoboxes.

**Depth**	**Coarse sand (%) 0.2–2.0 mm**	**Fine sand (%) 0.02–0.2 mm**	**Silt (%) 0.002–0.02 mm**	**Clay (%) <0.002 mm**	**Organic matter (%)**
0–0.2 m	46.4	39.7	5.5	7.0	1.4
0.2–2.0 m	26.8	51.1	4.2	17.6	0.3
2.0–4.0 m	21.1	54.1	8.7	16.0	<0.1

### Experimental Design

Oilseed rape (*Brassica napus* L., cv. “Butterfly”) plants were sown in the field on 16 August 2018 (Exp. 1) and in pots on 13 August 2019 (Exp. 2) before being transplanted to the rhizoboxes on 8 October 2018 and 26 August 2019, respectively. Due to a pest infestation *(Delia radicum)* in September 2019, a few plants were replaced by spare ones on 24 September 2019. The re-transplanted plants were smaller than the original ones during the entire growing period. Plant density in both years was five plants per chamber, corresponding to 14 plants m^−2^. In both experiments, each chamber was a replicate.

In Exp. 1, two N treatments were established by fertilizing the chambers with nutrient solution. Each chamber received either a high N treatment (N240) equivalent to 240 kg N ha^−1^, or a low N treatment (N80) equivalent to 80 kg N ha^−1^ on 27 March 2019. During this season, all chambers received water through precipitation and irrigation, which were sufficient to keep them well watered. In Exp. 2, two irrigation regimes were established. Rainout shelters were mounted on the top of all chambers on 26 February 2020, to allow complete control of soil moisture by irrigation. Well-watered (WW) chambers were irrigated with 60 mm water on 14 April and again on 15 April 2020 to establish soil profiles with high initial water content. No more irrigation was given to the well-watered chambers until 10 May 2020. In the following month, the well-watered chambers were irrigated frequently to keep an adequate water supply. Water-deficit (WD) chambers received no irrigation during the whole experimental period. In Exp. 2, all chambers were fertilized with a total of 200 kg N ha^−1^. Fertilization was divided into three applications, with N supplies of 40, 80, and 80 kg N ha^−1^ on 5 September 2019, 2 March, and 1 April 2020. The treatments and timeline of the experiments are shown in Table 2. The two treatments in Exps. 1 and 2 were established in six randomly distributed replicates.

**Table 2:** Treatments and timeline of the experiments.

	**Exp. 1**	**Exp. 2**
	**2018/2019**	**2019/2020**
Sowing	16 August 2018	13 August 2019
Transplanting	8 October 2018	26 August 2019^a^
Rate of N application	N240: 240 kg N ha^−1^; N80: 80 kg N ha ^−1^	200 kg N ha^−1^
Date of N application	27 March	1^st^: 5 September 2019; 2^nd^: 2 March; 3^rd^: 1 April
Water status	WW: Well-watered	WW: Well-watered; WD: Water deficit
Key irrigation events^b^	None	WW 1^st^: 14 April, 20 mm; WW 2^nd^: 15 April, 40 mm; WD: None
Tracer injection	3 April	17 April
Isotope sampling	1^st^: 2 April	1^st^: 16 April
	2^nd^: 10 April	2^nd^: 22 April
	3^rd^: 17 April	3^rd^: 27 April
	4^th^: 23 April	4^th^: 3 May
	5^th^: 1 May	5^th^: 8 May
Final biomass collection	5 June	18 June

*The oilseed rape plants were fertilized with 240 kg N ha^−1^ (N240) or 80 kg N ha^−1^ (N80) applications in Exp. 1; in Exp. 2, plants were grown under well-watered (WW) or water-deficit (WD) conditions at the beginning of the labeling*.

a *Pest infestation (Delia radicum) occurred in some of the chambers in September 2019. Thus, a few plants were replaced on 24 September 2019. Replaced plants were sown together with the original ones, but appeared smaller than the original ones throughout the experiment*.

b *Irrigation events that might change the soil water status before tracer injection were counted*.

### ^2^H and ^15^N Labeling

In both experiments, water and N uptake were traced using isotope labeled water and N injected into the soil at either 0.5- or 1.7-m depths. Each chamber received the labeled solution at only one depth. Tracer application was repeated in three chambers for each depth and treatment. Tracers were injected when the roots had already reached 1.7-m depth. The tracer application rates aimed at ensuring significant enrichment in plants and transpiration water and were based on estimated N and water availability in the soil, the natural isotope enrichment, and assumed uptake rates of applied tracers. In Exp.1 where two N fertilizer levels were established (240/80 kg ha^−1^), the ^15^N application was adjusted similarly and each chamber of the N240 and N80 treatments received 0.17 and 0.06 g ^15^N, respectively, equivalent to supplements of 4.8 and 1.6 kg N ha^−1^. In Exp. 2, each chamber received 0.09 g ^15^N, equivalent to fertilization of 2.5 kg N ha^−1^. Tracer solution was prepared by mixing the specific amount of Ca(^15^NO_3_)_2_ (>98.9 at% ^15^N) with 50 ml ^2^H_2_O (^2^H content = 99.94%) and 50 ml distilled water for each chamber.

The tracer was injected into 20 injection holes at each injection depth, which were evenly distributed in two parallel rows. The holes were made by a 30-cm-long steel stick that was 0.5 cm in diameter. Inside each of the 20 holes, a 5 ml of tracer solution was injected. The syringe needle was pushed 25 cm into the soil, and 1 ml of the solution was released every 5 cm as the syringe was drawn back. In this way, the tracer solution was distributed into 100 individual points in the soil at each injection depth. For further details on the labeling process, refer to Chen et al. (2021). The injection procedures were conducted between 1:00–4:00 pm on 3 April 2019 and 17 April 2020.

### Sampling and Sample Preparation

Transpiration water for ^2^H tracing and leaf samples for ^15^N tracing were collected five times in each experiment. The first sampling time was in the morning, right before the injection, and subsequently four times after the injection (Table 2). The collection of transpiration water was initiated between 10:00 and 11:00 on the sampling day. Each plant was covered with a plastic bag that was tightened by a rubber band at the bottom. After 2 h, the condensed droplets of transpired water inside the bags were collected. The water was quickly transferred from the bags to sealed plastic bottles. The collected transpiration water was filtered through 2-μm filter paper to remove dirt and debris. Filtered water from all plants grown in the same chamber was mixed for ^2^H analysis. Then, three to five of the latest fully developed leaves were collected on the same days as the transpiration water samplings. Leaf samples were dried, weighed, milled, and then encapsulated for ^15^N analysis. Since plant δ^13^C was reported to increase under water-limited conditions (Yousfi et al., 2012), the leaf samples were analyzed for ^13^C in Exp. 2 to determine the effect of water deficit on plant growth.

The total aboveground biomass was collected on 5 June 2019 and 18 June 2020, in Exps. 1 and 2, respectively. Biomass samples were divided into stems, pods, and leaves. However, in Exp. 2, all leaves had been shed when the total biomass was collected. Biomass samples from all plants in each chamber were mixed, dried at 70°C to constant weight, and weighed and stored until further analysis. In both experiments, biomass samples were analyzed for ^15^N.

Soil samples from 0.5-, 1.1-, and 1.7-m soil depths were taken before tracer injection and after the last isotope sampling to determine soil nitrate and ^15^N concentration. All soil samples were frozen immediately after sampling and stored until further preparation. Subsequently, 20 g soil was taken from each sample and mixed with 100 ml of 2M KCl solution. The mixture was shaken for 1 h and filtered through 2-μm filter paper. All solution samples were frozen for later analysis.

### Isotopic Analyses

All isotopic measurements were taken by the Stable Isotope Facility, UC Davis. ^15^N and ^13^C values in biomass samples were analyzed using IRMS. ^2^H values in transpiration water samples were analyzed using the Laser Water Isotope Analyzer V2 (Los Gatos Research, Inc., Mountain View, CA, USA). ^15^N concentration in soil samples was measured using IRMS. Nitrate-N content in the frozen soil solution was measured using the flow injection analyzer method.

To evaluate the dynamics of root water and N uptake during the labeling period, ^2^H and ^15^N enrichments (‰) were calculated as the increase of ^2^H and ^15^N values from pre-tracer sampling to post-tracer sampling unless otherwise stated. The ratio (%) of 1.7-m- and 0.5-m-derived ^2^H enrichment in transpiration water in the same treatment was calculated to investigate the distribution of water uptake. To compare ^15^N uptake between different treatments more directly, ^15^N uptake efficiency (^15^N_upe;_ % g ^−1^) was calculated as follows:


(1) 15Nupe=x(N15)sample− x(N15)controlN15a

where *x*(^15^N)_sample_ and *x*(^15^N)_control_ are the atom fraction of ^15^N in post-tracer samples and pre-tracer samples, respectively. In harvest samples, *x*(^15^N)_control_ refers to the natural abundance of ^15^N in plant organs, which is usually 0.366%. ^15^N_a_ is the total amount (g) of ^15^N that was added to the soil.

### Soil Water Measurements

A total of four time-domain reflectometry sensors (TDR-315/TDR-315L, Acclima Inc., Meridian, Idaho) were installed in each chamber. They were placed at 0.5-, 1.4-, 2.3-, and 3.5-m depth, respectively, recording soil volumetric water content (VWC; %) at least every 30 min. The VWC sensor readings were calibrated against VWC in soil samples taken in close proximity to the sensors. The samples were taken using metal rings with a diameter and height of 5 cm, and VWC was calculated based on the fresh and dry weight of the samples. For each sensor, at least three samples were collected at different times aiming to cover a broad range of water content. Based on the correlations between sensor VWC and sample VWC, the sensor readings were adjusted to obtain an intercept of zero. The correlations did not call for a slope adjustment.

The two periods around the middle of the isotope sampling period were selected for estimating the water uptake during the sampling period. In Exp. 1, it was a 20-day period starting from 4 days after injection and ending 4 days before the last sampling date. In Exp. 2, a 14-day period was selected, which began 4 days after injection and ended 4 days before the last sampling date. Letting each sensor represent a 1-m depth interval, the soil water content in each interval was calculated (mm m^−1^ soil column). No heavy water input was added during the selected periods; thus, soil water movement was assumed negligible, and a decrease in soil water content was interpreted as plant water uptake (Rasmussen et al., 2020). In both experiments, the daily water uptake was calculated only for the top 3-m soil columns, where most roots were found.

### Root Imaging, Segmentation, and Calculation

During the experimental periods, the growth of oilseed rape roots was recorded every 3–4 weeks with a digital camera (Olympus Tough TG 860). The camera was in a box excluding daylight but with internal LED light strips as the light source. The box fits the frames of each panel of the rhizobox chambers, and by taking five photos per panel, the total area of each panel was photographed for subsequent image segmentation. RootPainter (Smith et al., 2020a) was used to segment roots from the soil background. A model trained with randomly selected images was used to segment roots on all the images and estimate the root length in each image *via* skeletonization and pixel counting (Smith et al., 2020b). Root intensity was calculated as cm of root per cm^2^ of soil in the images.

### Statistics

Data analyses were conducted in R (Version 3.5.3, R Core Team, 2019). The effect of N and water treatment on the harvested biomass and N content was tested in *t*-tests in separate tests for each experiment and each plant organ. The *t*-tests were used for comparing root intensity under different treatments. Separate tests were performed for each experiment and depth. For both experiments, the means of daily water uptake between treatments at each depth were compared by *t*-tests. In Exp.2, the effects of water treatments on foliar δ^13^C were tested in a two-way repeated measurements ANOVA with water treatment and date as factors. Linear mixed models were used to examine the differences in ^2^H enrichment in water samples and ^15^N_upe_ in biomass samples among N/water treatments, dates, and injection depths, where the combined factor of N/water level and depth (level-depth combined treatment, e.g., N80 - 0.5 m) and dates were fixed effects and chamber was a random effect. Multiple comparisons were conducted subsequently to test for changes in ^2^H enrichment and ^15^N_upe_ within the same level-depth combined treatment among all the dates. In both experiments, the effect of N/water supply on ^15^N_upe_ in harvest samples within the same organ was tested using linear mixed models with level-depth combined treatment as a fixed factor and chamber direction as a random factor. Multiple comparisons were done to compare ^15^N_upe_ in harvest samples within the same organ.

For ^2^H enrichment analysis, data were log-transformed to fulfill assumptions of normality and homogeneity. Multiple comparisons (Tukey's honestly significant difference (HSD); *p* ≤ 0.05) were based on the values derived from linear mixed models.

## Results

### Biomass

Oilseed rape plants grew well in both years. In Exp. 1, the effect of N fertilization rate was evident, as the N240 treatment resulted in significantly higher leaf, stem, and pod biomass than N80 (Table 3). The N content in all three organs also increased when more N was given.

**Table 3:** Mean plant dry matter and N contents for different N or water treatments at final collection (*n* = 6).

**Experiment**	**N level**	**Water status**	**Plant biomass (g chamber** ^**−1**^**)**			**N content (mg g** ^**−1**^ **)**		
			**Leaf**	**Stem**	**Pod**	**Leaf**	**Stem**	**Pod**
Exp. 1	N240	WW	56.2a	299.7a	281.5a	18.3a	7.1a	17.4a
	N80	WW	31.9b	215.7b	179.4b	17.1b	5.7b	16.3b
Exp. 2	N200	WW	^*^	357.7a	445.5a	^*^	3.7a	11.1a
	N200	WD	^*^	326.5a	410.9a	^*^	3.9a	11.8a

*In Exp. 1, the oilseed rape plants were grown under 240 kg N ha^−1^ (N240) or 80 kg N ha^−1^ (N80) applications and were all well-watered (WW); in Exp. 2, all plants were fertilized with 200 kg N ha^−1^ and grew under well-watered or water-deficit (WD) conditions. In the same column, different letters indicate significant differences between treatments in Exp. 1 (p < 0.05). No significant differences were found between water treatments in Exp. 2. Pods were not fully ripe in either experiment*.

* *There were no leaves left when oilseed rape plants were harvested in 2020*.

No significant differences were found in biomass or N content between the water treatments in Exp. 2. Plants that grew under lower soil water content tended to have a lower stem and pod biomass, whereas the N content in the pod and stem samples at harvest was slightly higher when less water was supplied, although not significant (Table 3).

### Root Growth

**Figure 1:**
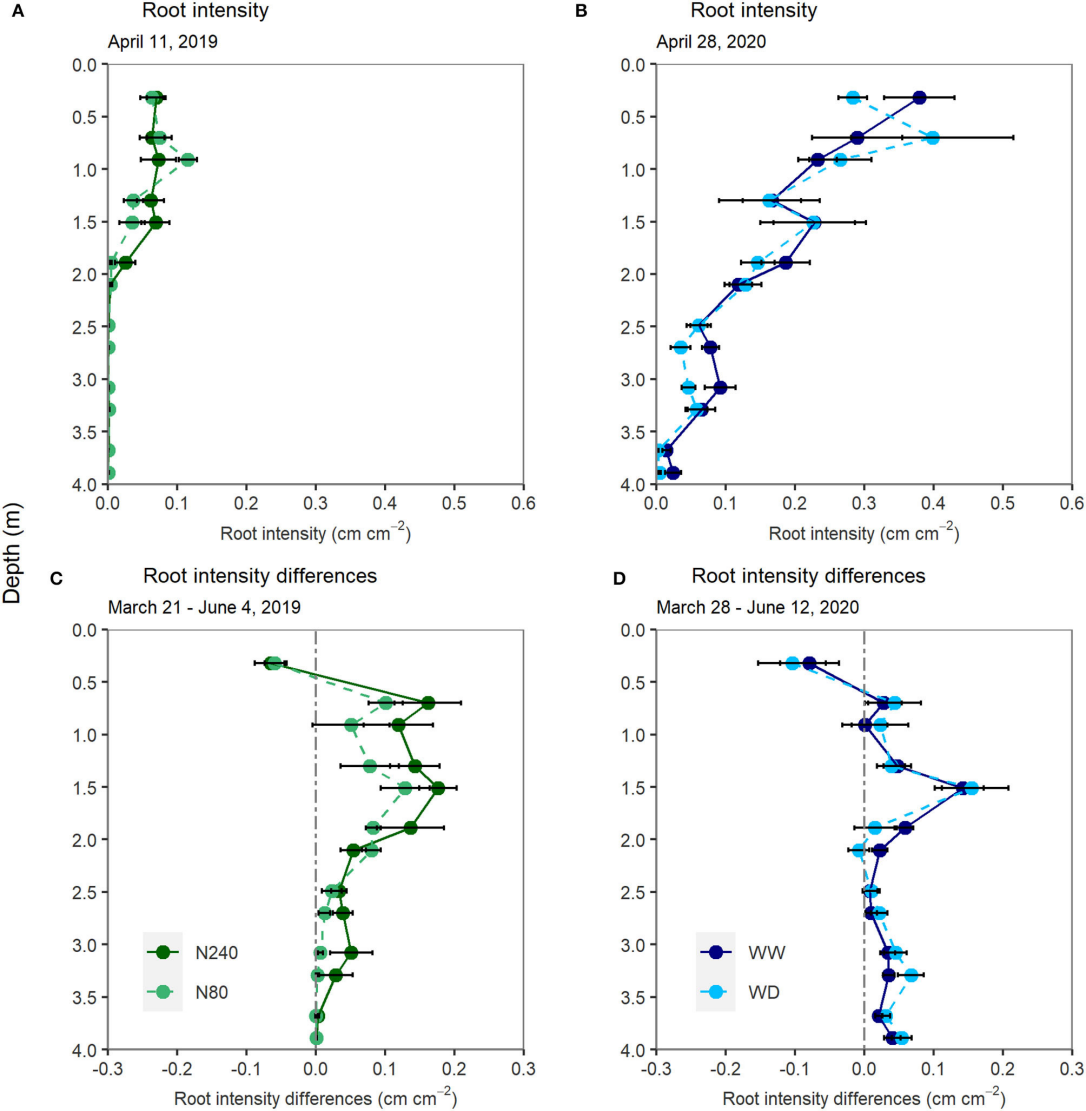
Root intensity measured on 11 April 2019 **(A)**, 8 days after tracer injection and on 28 April 2020 **(B)**, 11 days after tracer injection. Differences in root intensity from 21 March to 4 June 2019 **(C)** and from 28 March to 12 June 2020 **(D)**. N240 = 240 kg N ha^−1^, N80 = 80 kg N ha^−1^, WW = well-watered, WD = water deficit. Error bars denote standard errors (*n* = 6). No significant differences were found at any depths.

Root growth was recorded from March to June, covering tracer injection and sampling periods in both years (Figure 1). Roots were present below 1.7 m already in April in both experiments. In Exp. 1, roots reached just below 2-m depth during the labeling period (Figure 1A). In Exp. 2, roots were present below 3 m in April (Figure 1B). At the time of labeling, the average root intensities in the top 2-m soil layers were approximately four times higher in Exp. 2 than in Exp. 1 (0.25 and 0.06 cm cm^−2^, respectively).

In both years and all treatments, root intensity tended to increase below 0.5 m from March to June (Figures 1C,D). There was a tendency toward more root growth in the N240 than in the N80 treatment in the lower soil layers. No significant differences in root growth were found between the two water regimes. In both experiments, the root intensity in the top 0.5 m decreased from March to June.

### Water Extraction

**Figure 2:**
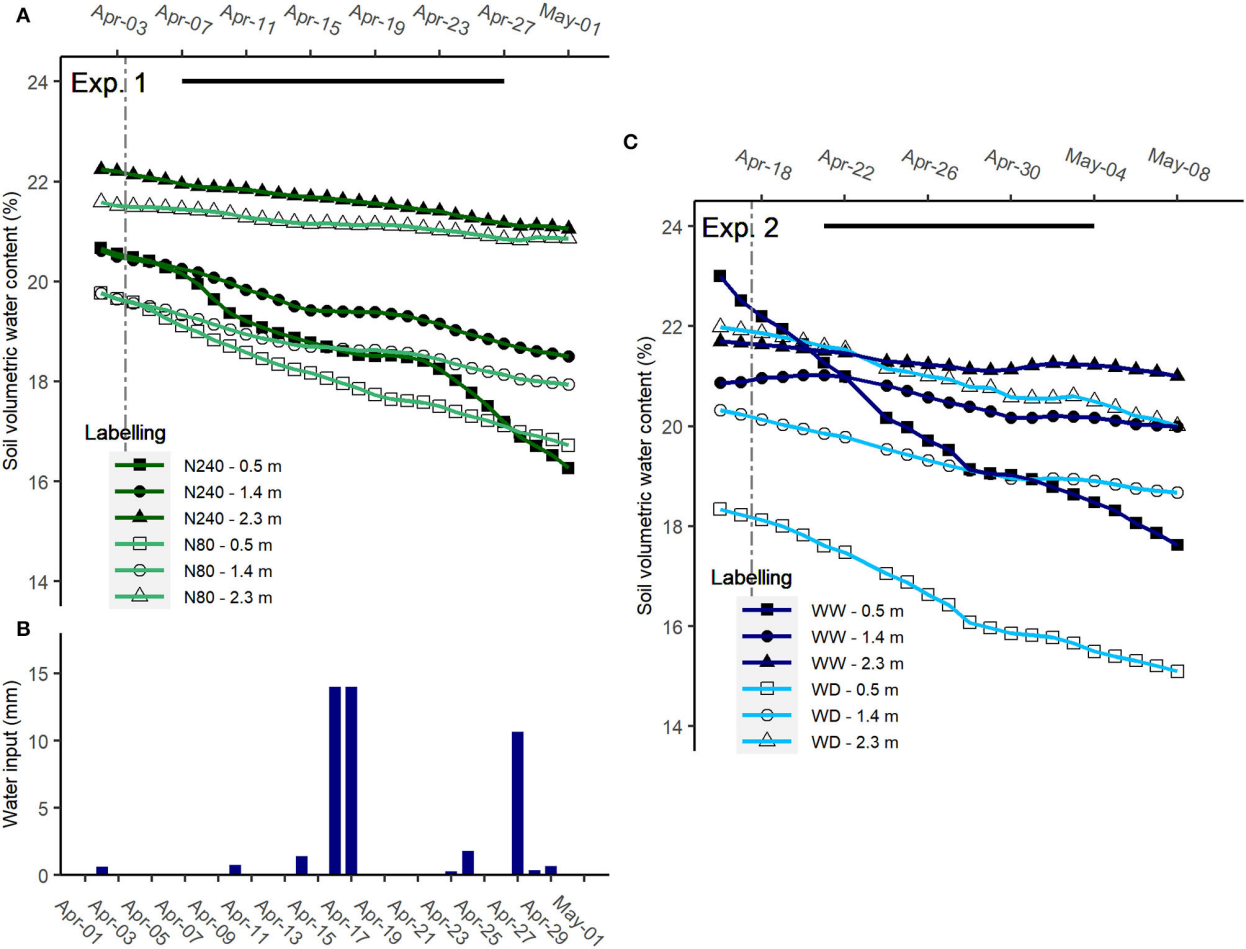
Soil volumetric water content [**(A)**, VWC; %] and total water input [**(B)**, irrigation + precipitation, mm] in Exp. 1 and soil VWC in Exp. 2 **(C)**. N240 = 240 kg N ha^−1^, N80 = 80 kg N ha^−1^, WW = well-watered, WD = water deficit. Data were collected from 2 April to 1 May 2019 in Exp. 1 and from 16 April to 8 May 2020 in Exp. 2. Daily averages of recorded VWC are shown in **(A,C)**. The black segments denoted selected periods for daily water decrease estimations in Exp. 1 and Exp. 2, refer to Figure 3.

Volumetric water content at the three recorded depths was similar in the two N treatments during the isotope sampling period in Exp. 1 (Figure 2A). To avoid drought stress in surface layers, extra water was given occasionally during the labeling period (Figure 2B). Extra water inputs, especially irrigation events on 18 and 19 April 2019, slowed down the decrease of soil VWC in the following 2 days. In Exp. 2, where no water was given during the sampling period, only the VWC at 0.5-m depth in the water-deficit treatment tended to be lower (Figure 2C).

**Figure 3:**
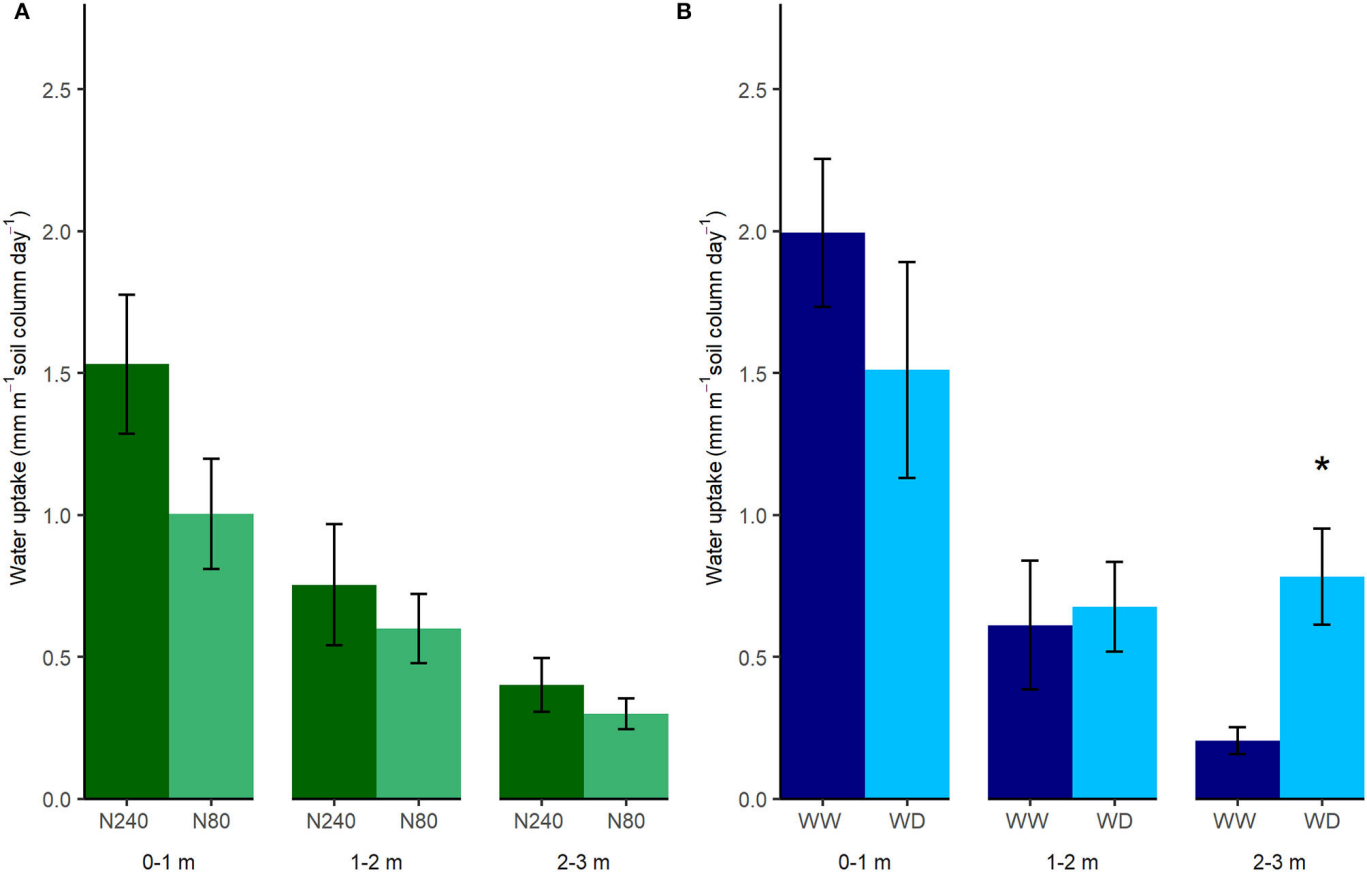
Estimated daily water uptake in Exp. 1 **(A)** and Exp. 2 **(B)**. N240 = 240 kg N ha^−1^, N80 = 80 kg N ha^−1^, WW = well-watered, WD = water deficit. Daily water uptake from each 1 m interval of soil column was estimated as averages of daily water decrease from that column from 7 April to 27 April 2019 in Exp. 1 and from 21 April to 4 May 2020 in Exp. 2. Error bars denote standard errors (*n* = 6), and stars indicate significant differences between the treatments at the same depth in the same experiment (*p* < 0.05).

Based on the simplified estimations of daily water uptake, more than 1 mm of water was removed from the 0–1-m soil layer per day during the selected labeling period, while <1 mm was removed from the 1–2- and 2–3-m soil layers in Exp. 1 (Figure 3A). It was clear that with higher N application, water uptake throughout the whole soil profile increased, though not significantly. In the high N treatment, daily water uptake from 0–1, 1–2, and 2–3 m was 1.53, 0.75, and 0.40 mm, respectively. In the low N treatment, the uptake from 0–1, 1–2, and 2–3 m was 1.00, 0.60, and 0.30 mm, respectively. With a high N supply, roots extracted more than 57.1% water from the top 1-m soil. In the low N treatment, this ratio was 52.6%.

**Figure 4:**
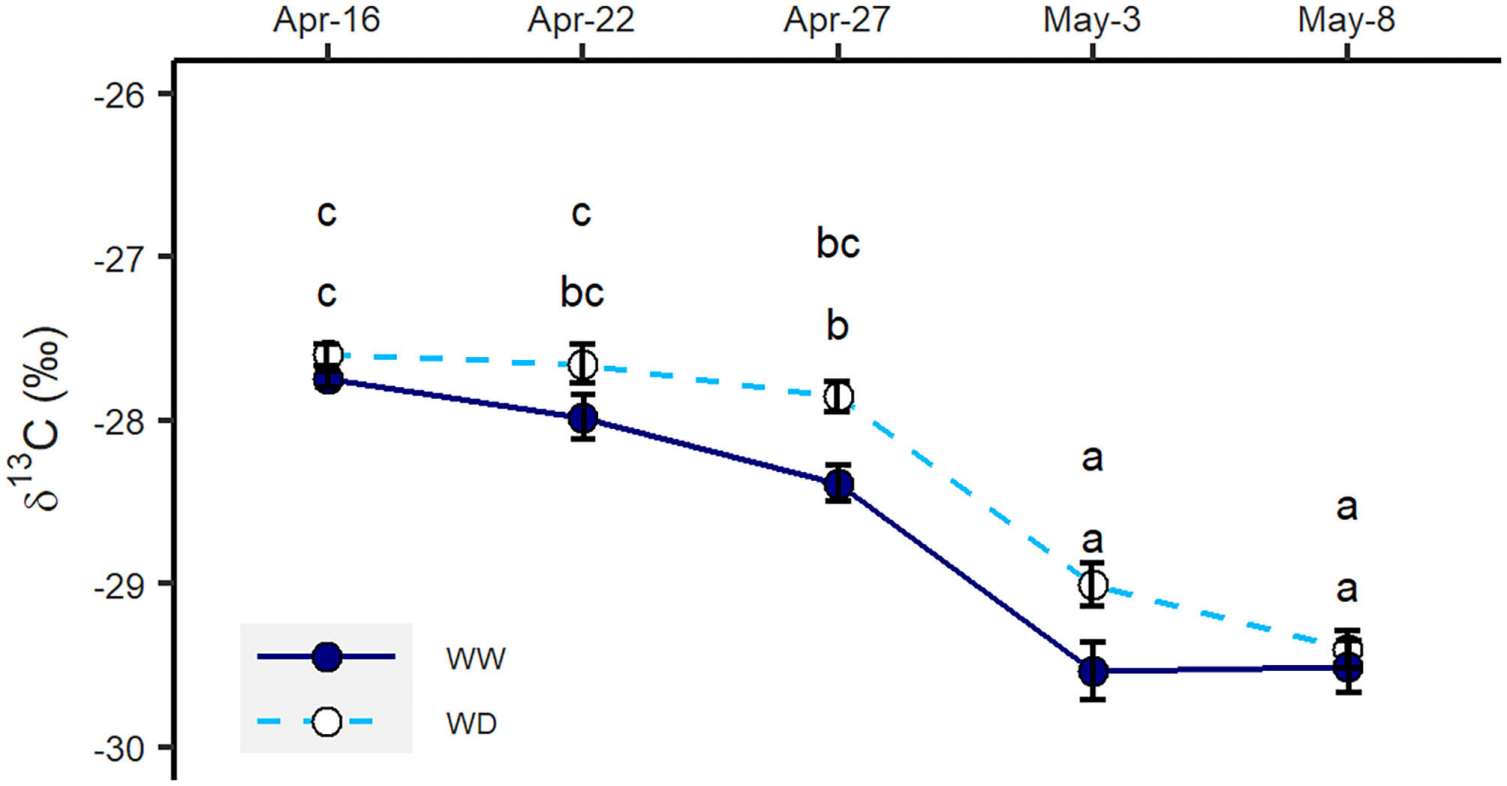
δ^13^C measured in leaf samples collected during the isotope sampling period in Exp. 2 in well-watered (WW) and water-deficit (WD) treatments. Error bars denote standard errors (*n* = 6), and letters indicate significant differences across the treatments (*p* < 0.05).

The total amount of water taken up in the two water regimes in Exp. 2 was similar. In total, 2.80 and 2.97 mm water per day were removed within the selected period from the top 3 m of the soil column in the WW and WD treatments, respectively (Figure 3B). Although roots took up less water from 0–2 m, a shift toward water uptake from deeper soil layers in the WD treatment was observed. In the 2–3-m interval, plants under the WD treatment took nearly four times the amount of water as the WW treatment (Figure 3B). However, slight increases in δ^13^C values indicated plants under the WD treatment were insignificantly drought-stressed during the labeling period in Exp. 2 (Figure 4).

### ^2^H Enrichment

**Figure 5:**
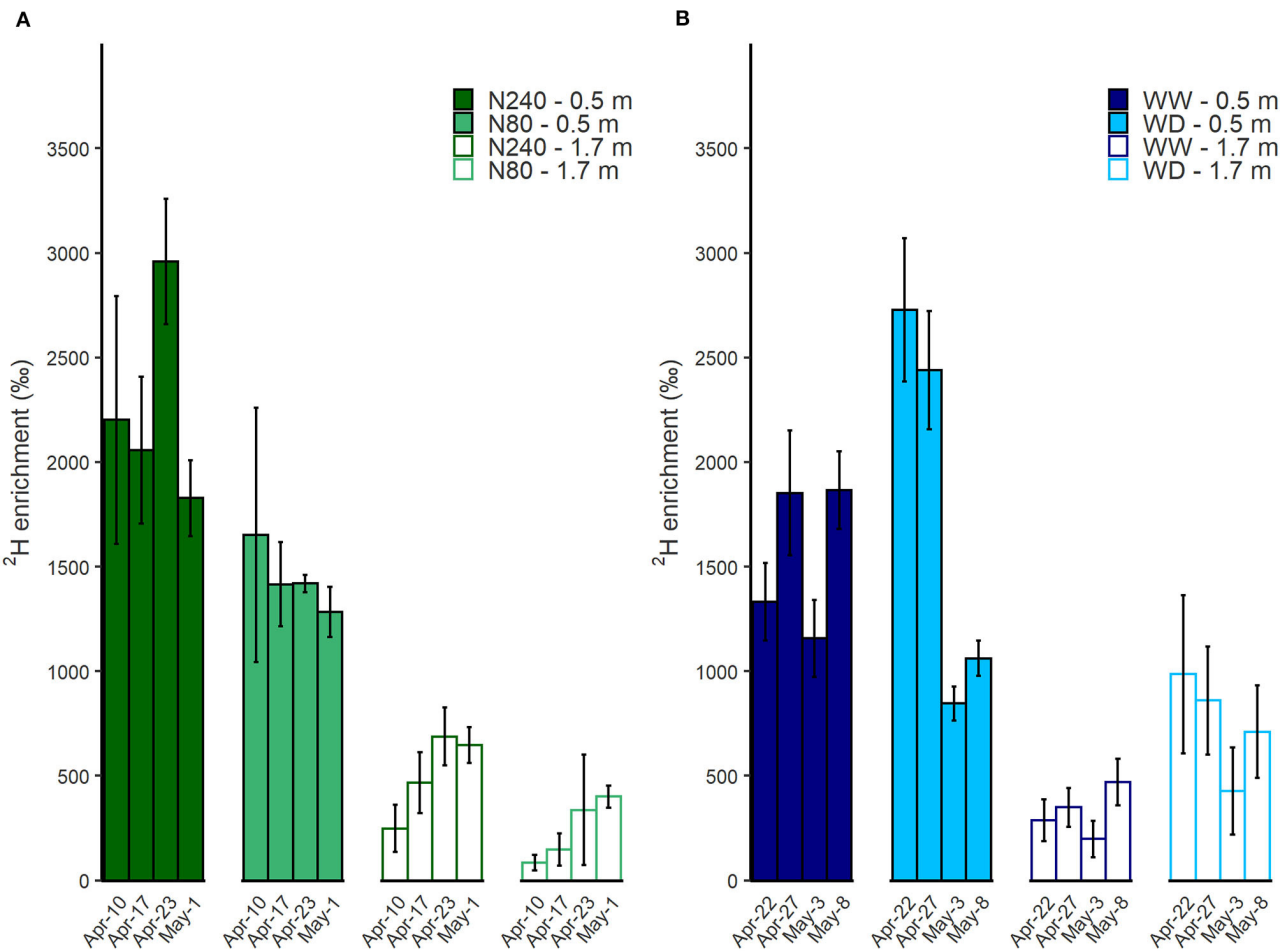
Time course of ^2^H enrichment in transpiration water was shown under N **(A)** or water **(B)** treatments during isotope sampling periods. N240 = 240 kg N ha^−1^, N80 = 80 kg N ha^−1^ **(A)**, WW = well-watered, WD = water deficit **(B)**. ^2^H labeled water was injected at either 0.5 or 1.7 m in each treatment. Error bars denote standard errors (*n* = 3). Mean values are shown here (± SE).

At both N treatments, when the tracer was injected at 0.5-m depth instead of 1.7 m, a higher enrichment of ^2^H in transpiration water was observed (Figure 5A). The ^2^H enrichment of the transpiration water was higher in N240 than in N80 treatments on all dates and both injection depths. The concentration of ^2^H in transpiration water increased significantly with time when the injection was conducted at 1.7 m. However, when ^2^H was injected at 0.5 m, no increase in concentration with time was observed.

During the labeling period in Exp. 2, the lowest ^2^H concentration in the transpiration water was found in the WW treatment when the tracer was injected at 1.7 m. Higher ^2^H concentrations were observed in the WD treatment than the WW treatment for the first sampling dates. In the WD treatment, ^2^H concentration then fell by c. 60% between 27 April and 3 May, while the WW treatment ^2^H level remained consistent (Figure 5B).

In Exp. 1, the enrichments of ^2^H derived from 1.7 m were 5–35% of the ^2^H derived from 0.5 m in both treatments, showing a larger proportion of ^2^H uptake from 0.5 m than 1.7 m (Table 4). For Exp. 2, in the WW treatment, the ratio of ^2^H enrichment with tracer injected at 1.7 and 0.5 m was approximately 20% in 3 weeks after injection. While in the WD treatment, the ratio was over 30% and further increased by 30% points at the last sampling date (Table 4).

**Table 4:** The ratio of ^2^H – enrichment in transpiration water with tracer injected at 1.7 m (Enrich_1.7_) and 0.5 m (Enrich_0.5_).

**Experiment**	**Year**	**N level**	**Water status**	**Sampling date**	**Enrich_**1.7**_/ Enrich_**0.5**_ (%)**
Exp. 1	2019	N240	WW	10 April	11
				17 April	23
				23 April	23
				1 May	35
		N80	WW	10 April	5
				17 April	10
				23 April	24
				1 May	31
Exp. 2	2020	N200	WW	22 April	22
				27 April	19
				3 May	17
				8 May	25
		N200	WD	22 April	36
				27 April	35
				3 May	50
				8 May	67

*The oilseed rape plants were grown under 240 kg N ha^−1^ (N240) or 80 kg N ha^−1^ (N80) applications and were all well-watered (WW) in Exp. 1; in Exp. 2 all plants were fertilized with 200 kg N ha^−1^ fertilizer, and grew under well-watered or water-deficit (WD) conditions*.

### N Depletion and Accumulation

In spring both years, soil nitrate concentrations of the top 1.7-m soil were low and did not change much between the first and second sampling dates (Table 5). Compared with Exp. 1, split fertilization in Exp. 2 allowed more robust plant growth at the early growth stage. Denser and deeper roots were more efficient at exploiting soil available N. Therefore, soil N and ^15^N concentrations were even lower in Exp. 2 than in Exp. 1. In Exp. 1, there was a high variation in ^15^N concentration in the soil nitrate at the soil sampling after the isotope sampling period, and no significant differences were found between nitrate and ^15^N under different N treatments at any of the sampled depths. In Exp. 2, no significant differences were found in nitrate depletion between the WW and WD treatments (Table 5).

**Table 5:** Means of soil nitrate content and ^15^N concentration in different soil layers before and at the end of labeling periods (in 0.5 and 1.7 m, *n* = 3; in 1.1 m, *n* = 6).

**Experiment**	**N level**	**Water status**	**Depth (m)**	**Soil ${\text{NO}}_{3}^{-}$before (mg N kg^**−1**^ dry weight)**	**Soil ${\text{NO}}_{3}^{-}$after (mg N Kg^**−1**^ dry weight)**	**Soil ^**15**^N before (‰)**	**Soil ^**15**^N after (‰)**
Exp. 1	N240	WW	0.5	2.6	2.0	102	1,116
			1.1	2.4	2.2	19	33
			1.7	2.9	2.2	163	8,669
	N80	WW	0.5	2.4	1.7	364	1,323
			1.1	1.8	2.4	31	38
			1.7	2.9	2.9	70	5,109
Exp. 2	N200	WW	0.5	0.1	0.1	^*^	^*^
			1.1	0.1	0.2	^*^	^*^
			1.7	0.1	0.2	^*^	^*^
	N200	WD	0.5	0.1	0.1	^*^	^*^
			1.1	0.1	0.1	^*^	^*^
			1.7	0.1	0.1	^*^	^*^

*The oilseed rape plants were grown under 240 kg N ha^−1^ (N240) or 80 kg N ha^−1^ (N80) applications and were all well-watered (WW) in Exp. 1; in Exp. 2, all plants were fertilized with 200 kg N ha^−1^ fertilizer and grew under well-watered or water-deficit (WD) conditions. In Exp. 1, soil samples were taken on 3 April and 3 May 2019; in Exp. 2, soil samples were taken on 17 April and 9 May 2020. For soil nitrate content analysis, soil samples, which were taken from labeled depths, were excluded to avoid the effects of tracer application. For soil ^15^N concentration analysis, soil samples of 0.5 and 1.7 m were taken from labeled depths, soil samples of 1.1 m were taken from all depths*.

* *Soil ^15^N concentration was too low to detect*.

**Figure 6:**
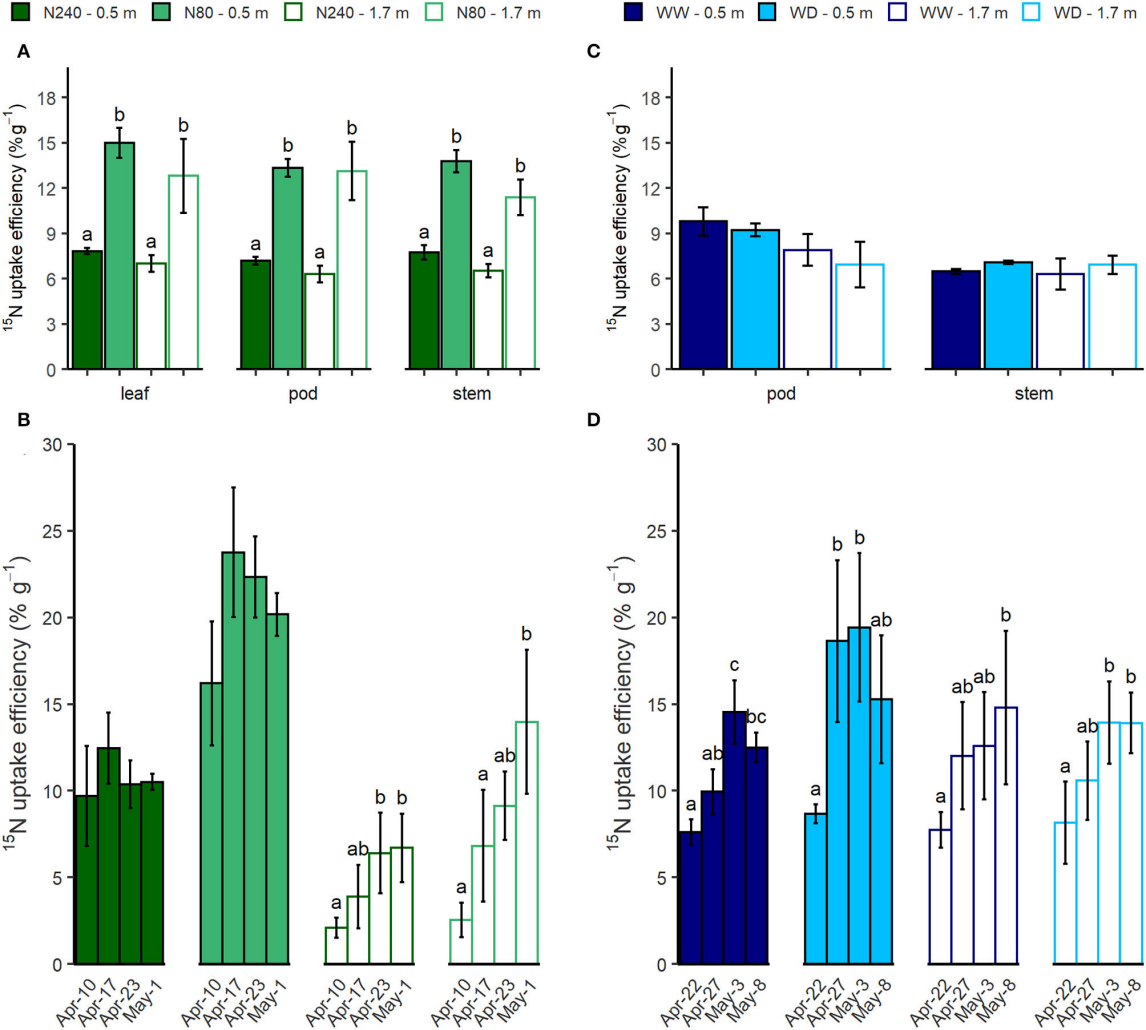
^15^N uptake efficiency (% g^−1^) that was measured in harvest samples **(A,C)**, and leaf samples **(B,D)** which were collected during the labeling periods in Exp. 1 **(A,B)** where N240 = 240 kg N ha^−1^, N80 = 80 kg N ha^−1^ and Exp. 2 **(C,D)** where, WW = well-watered, WD = water deficit. ^15^N tracer was injected at either 0.5 or 1.7 m in each treatment. Mean values of harvest ^15^N use efficiency in different organs under different N **(A)** or water **(B)** treatments are shown here (± SE). Error bars denote standard errors (*n* = 3), and letters indicate significant differences among treatments within the same organ (*p* < 0.05). Time course of leaf ^15^N use efficiency (% g ^−1^) under different N **(C)** and water **(D)** regimes was calculated. Error bars denote standard errors (*n* = 3), and letters indicate significant differences among all sampling dates under the same treatment and injection depth (*p* < 0.05). Mean values are shown here (± SE).

Corrected for ^15^N already in the soil before tracer injection, additional ^15^N tracer resulted in higher ^15^N enrichment in the biomass samples (Figure 6). At the cessation of Exp. 1, plants under the same N treatment, with ^15^N tracers injected at either 0.5 or 1.7 m, exhibited similar ^15^N uptake efficiency (Figure 6A). Compared with N240 treatment, plants in N80 treatment were ^15^N-labeled at the same concentration but with fewer doses. Additionally, oilseed rape plants in the N80 treatment had higher ^15^N use efficiency, which was two times as high as in the N240 treatment (Figure 6A). In general, an extra gram of ^15^N led to a 12–15% increase in biomass ^15^N atom fraction in the N80 treatment, whereas in the N240 treatment, the increase in ^15^N atom fraction per gram of ^15^N added was only around 6% (Figure 6A). During the labeling period in Exp. 1, the leaf ^15^N uptake efficiency tended to increase with time (Figure 6B), which indicated that more ^15^N had accumulated in the leaves. However, this was only significant when ^15^N was injected at 1.7 m. As independent of ^15^N injection depths, a larger fraction of the applied ^15^N was found in leaves of oilseed rape plants that had been fertilized with a lower amount of N.

^15^N uptake efficiency was almost unaffected by the soil water status or injection depth in the harvest samples of Exp. 2 (Figure 6C). Whereas the fraction of ^15^N in leaves significantly increased with time, no clear water treatment or injection depth effect was observed at any measurement date (Figure 6D).

## Discussion

### Effects of N and Water Supply on Plant Biomass and Root Growth

There is no doubt that supplemental N and water supply affect biomass production. In general, oilseed rape plants grown with extra N and water supply have been shown to have a higher yield (Taylor et al., 1991; Schjoerring et al., 1995; Dresbøll et al., 2016; Riar et al., 2020). In this study, a higher fertilization rate exhibited positive effects on biomass and plant N content (Table 3). However, we only observed slight and non-significant increases in biomass under the well-watered conditions, together with a non-significant decrease in plant N content (Table 3). This showed that the overall growth and development of oilseed rape plants were not restricted by water deficiency.

The difference in root growth along the soil profile between two N treatments was small in this study (Figure 1). However, we found a tendency toward deeper and denser roots in the subsoil after the high rate of N application. The effects of N supply on root growth were found to be inconsistent with previous studies. Svoboda and Haberle (2006) claimed that a high N fertilization rate led to reduced wheat rooting depth and reduced root density in deeper layers, whereas Hodge et al. (1999) found increased soil N availability led to stronger shoot and root growth. In this study, although root intensity and rooting depth did not remarkably increase with N supply, plant biomass in the high N treatment was significantly increased. This indicates that a higher allocation of assimilates to roots may exist in high N treatment, from which roots can supply more N to the shoots. As Lynch et al. (2012), pointed out, although root/shoot ratio decreased with N supply, higher branching with finer roots can increase the root surface area and maintain shoot nutrient supply.

According to the previous studies, water deficiency is a stimulator of deep root growth and the uptake of deep soil water (Bloom et al., 1985; Vandoorne et al., 2012). Surprisingly, the effect of water supply on root growth was not seen in this study. In both treatments, the increase in root intensity along the soil profile except at 1.5-m depth was <0.1 cm cm^−2^, indicating less or no preferential root growth under short-term water deficit. As indicated by foliar δ^13^C measurements (Figure 4), one possible explanation is that the water deficiency was not severe or long enough to stimulate the deep root growth observed in previous studies (Skinner, 2008; Vandoorne et al., 2012). Another explanation is that the plants under the water-deficit treatment were able to obtain adequate water from the subsoil. Therefore, their growth was not stimulated by the water deficit.

### Effects of N Supply on Deep Water Uptake

N supply affected the amount, distribution, and dynamics of water uptake. With a higher N fertilization rate, root water uptake was higher at all depths along the soil profile, with most of the water taken from the top 1-m layer where most of the roots were located and directly affected by the N supply (Figures 2A, 3A). Water uptake is promoted by N supply *via* increasing aboveground growth, transpiration, and photosynthesis (Taylor et al., 1991; Waraich et al., 2011). In addition, high nitrate supply has been found to improve radial water fluxes in roots, as it upregulates the expression of aquaporin and enhances root hydraulic conductivity (Gorska et al., 2008; Wang et al., 2016). Although it was not shown in our case, lateral root formation and the extension of main roots with high N supply (Drew et al., 1973) could also account for the increased water uptake in the topsoil. Higher ^2^H enrichment in transpiration water was found in N240 treatment compared to N80 treatment where the tracer was applied at 0.5 or 1.7 m (Figure 5A). This could be an indicator for a higher water uptake proportion from the labeled depth or shows that the total water uptake was lower (Rasmussen and Kulmatiski, 2021). In the current experiment, it indicated a larger fraction of water was taken from the labeled depth with the high N supply. Interestingly, with a low N supply, the fraction of overall deep-extracted water (below 1 m) was 4.5% higher than that with a high N supply (Figure 3). This may indicate the beneficial effect of reducing N supply on utilizing deep-stored water. Accessing and using deep-stored water is valuable to agricultural production, especially in dry areas (Lilley and Kirkegaard, 2016). However, one should notice that low N supply also reduces plant biomass (Table 3). It is important to find the balance between improving yield, maximizing deep-stored resource use, and minimizing N input.

### Effects of N Supply on Deep N Uptake

Increasing N fertilization from 80 to 240 kg N ha^−1^ did not significantly change the content of inorganic N in 0.5- or 1.7-m soil, and water deficiency had less effect on the concentration and distribution of soil nitrate. However, these findings should be interpreted with caution as there were only a few weeks between the applications of N or water treatments until the soil measurements. In Exp. 1, fertilizer was applied to the surface soil. Without heavy water input, nitrate would leach downward slowly. This may explain the less variation of subsoil N concentration during the labeling period (Table 5). Furthermore, although deeper roots contribute to total N uptake, most of the N uptake still occurs in topsoil. The relatively less N uptake from deeper soil layers may not significantly affect soil N concentration there. In other studies, when such water or N treatments lasted longer, oilseed rape grown under higher N fertilization rate or less water supply was observed to be left more soil nitrate in topsoil layers (Smith et al., 1988; Dresbøll et al., 2016).

N uptake efficiency depends on both root uptake capacity and crop demand. Oilseed rape needs high N input during the vegetative growing stage (Rathke et al., 2006). Nevertheless, even with a high capacity to absorb N in autumn and winter, the recovery of fertilized N by oilseed rape is generally found to be poor (Sieling and Kage, 2010), and an increasing rate of N supply can further reduce the N recovery (Rathke et al., 2006; Bouchet et al., 2016). This reduction in N recovery was also observed in our study. Despite higher biomass in the N240 treatment, ^15^N recovery in the biomass was higher in the N80 treatment (Figure 6A), indicating more complete soil N depletion under a lower N fertilization rate. Svoboda and Haberle (2006) pointed out that the effect of high nitrogen supply in the topsoil on reduction of N depletion in the subsoil can be the result of both less N demand from the subsoil and reduced root growth in the subsoil. In our case, the latter was not observed.

### Effects of Water Supply on Deep Water Uptake

The relationship between ^13^C isotopic composition or discrimination and water stress has been widely studied for evaluating the performance of crops under water stress (Farquhar and Richards, 1984; Farquhar et al., 1989; Dercon et al., 2006). Plant δ^13^C was reported to increase under water-limited conditions (Yousfi et al., 2012). In our second experiment, the foliar δ^13^C values showed that water deficiency did not severely stress the oilseed rape plants (Figure 4), indicating plants under WD treatment took adequate water to maintain their growth. Previous findings by Vandoorne et al. (2012) and Hashemian et al. (2015) showed that the distribution of root water uptake can be altered by the moisture level of different soil layers. Vandoorne et al. (2012) observed that chicory roots in the deeper soil layers were able to extract more water when topsoil was drought-stressed. However, Rasmussen et al. (2020) concluded that chicory failed in compensating water uptake from deeper soil layers when drought stress occurred in topsoil. They suggested that the high hydraulic resistance and drought-induced stomatal closure might reduce root water uptake and plant water demand, leading to the failure of compensation (Rasmussen et al., 2020). Our results show that oilseed rape plants are able to extract more water from deeper and wetter soil layers to compensate for the reduction of water absorption in shallower and drier layers (Figure 3B). In this way, water-deficit plants are able to maintain their growth and development.

### Effects of Water Supply on Deep N Uptake

Effects of water supply on ^15^N uptake efficiency were not observed in this study (Figures 6C,D). This contrasts with others' findings that water deficit reduces N uptake and use efficiency in various ways (Jensen et al., 1997; Gonzalez-Dugo et al., 2010; Sadras et al., 2016; Riar et al., 2020). In general, topsoil water deficit reduces the availability of soil N (Jensen et al., 1997) and restricts its movement *via* mass flow and diffusion (Plett et al., 2020), hence reducing N uptake from top layers. To meet crop demand, the N uptake from subsoil would be stimulated. However, this did not seem to be the case in our study. Several possible explanations may account for the absent observation of compensated N uptake. One is that the extent of the water deficit was not severe enough to reduce soil N availability and subsequent N uptake. In this case, plants are still available to take N from all depths. There is also a possibility that soil dryness directly reduces crop N demand *via* reducing the shoot growth; therefore, the efficient N uptake from subsoil was no longer needed. In the current setup, the fertilization rate was relatively high. Thus, a likely explanation is that the applied N was excess plant demand, where compensation from deeper soil layers was not required. Alternatively, there may exist N compensation from deeper soil layers, whereas the resolution of the isotope analyses was not sufficient to measure any possible compensation.

### Deep Roots for Water and N Uptake

Our results also demonstrate the rising importance of deep roots for water uptake in the late growing periods. During the labeling period, a larger fraction of water was taken by roots in the deeper soil layers. Except in the WW treatment, the ratio of 1.7 m to 0.5 m derived ^2^H-enrichment increased with time. The increments were 24, 26, and 31% points in N240, N80, and WD treatment, respectively (Table 4). The effect of time on deep water uptake may be due to a direct effect on the development and maturity of roots, which has also been demonstrated by Garrigues et al. (2006), or to exhaustion of the labeled ^2^H at the 0.5-m depth.

Still, ^15^N uptake was less affected by deep resource placement than ^2^H uptake during the 3-week sampling period (Figures 5, 6). The injection depth did not affect ^15^N uptake efficiency measured in harvest biomass samples in either experiment (Figures 6A,C), whereas the continuous leaf sampling after injection showed different patterns of ^15^N uptake dynamic at 0.5 and 1.7 m (Figures 6B,D). When ^15^N-labeled nitrate was applied at 0.5 m, the ^15^N uptake efficiency reached a peak in 1 to 2 weeks, whereas the efficiency kept increasing after injection at 1.7 m, suggesting some ^15^N-labeled nitrate still remained in the soil (Figures 6B,D). Besides, in Exp. 1, 1 month after the injection, soil ^15^N enrichment at 1.7 m was much higher than at 0.5 m, which further confirmed that more ^15^N had been left at 1.7 m after injection (Table 5). The continuous and delayed N uptake from subsoil was also observed in winter wheat by Haberle et al. (2006). They suggested that the inadequate N supply from topsoil might be the stimulator of subsoil N uptake. The differences in ^2^H and ^15^N uptake patterns indicate that while we see substantial uptake of both water and N by the deep parts of the root system, uptake of the two resources is not equally limited by the low root density and the short time available for active uptake (Chen et al., 2021).

## Conclusions

Overall, when roots are already well developed in upper soil layers, N and water supply could still alter the water and N uptake *via* regulating deep water and N acquisition. An established deep root system was able to compensate for the reduction of water uptake in dry topsoil when water stress occurred. Our results highlight the rising importance of deep roots in ensuring adequate water uptake in the late growing season. Our study shows that plants with low N supply have more complete N utilization, which mitigates the risk of N leaching from the subsoil. Our results also highlight the potential benefits of reducing N supply for utilizing deep-stored water. However, the cost might be the loss of biomass. Further research on optimal water and N management strategies and their effects on deep root function is required to maximize the benefits of deep roots and maintain biomass production.

## Data Availability Statement

The original contributions presented in the study are included in the article/supplementary material, further inquiries can be directed to the corresponding author/s.

## Author Contributions

KT-K, DD, and GC initiated the research and designed the experiment. GC carried out the experiment and wrote the manuscript with support from KT-K and DD. CR and AS helped with data analysis and manuscript revision. All authors contributed to the article and approved the submitted version.

## Funding

This study was supported by Villum Foundation (DeepFrontier project, grant number VKR023338). The China Scholarship Council provided financial support for GC for her Ph.D. research.

## Conflict of Interest

The authors declare that the research was conducted in the absence of any commercial or financial relationships that could be construed as a potential conflict of interest.

## Publisher's Note

All claims expressed in this article are solely those of the authors and do not necessarily represent those of their affiliated organizations, or those of the publisher, the editors and the reviewers. Any product that may be evaluated in this article, or claim that may be made by its manufacturer, is not guaranteed or endorsed by the publisher.

## References

[B1] Álvarez S. Navarro A. Nicolás E. Sánchez-Blanco M. J. (2011). Transpiration, photosynthetic responses, tissue water relations and dry mass partitioning in Callistemon plants during drought conditions. Sci. Hortic. (Amsterdam). 129, 306–312. 10.1016/j.scienta.2011.03.031

[B2] Asare E. Scarisbrick D. H. (1995). Rate of nitrogen and sulphur fertilizers on yield, yield components and seed quality of oilseed rape (*Brassica napus* L.). F. Crop. Res. 44, 41–46. 10.1016/0378-4290(95)00051-7

[B3] Bloom A. J. Chapin F. S. Mooney H. A. (1985). Resource limitation in plants - an economic analogy. Annu. Rev. Ecol. Syst. Vol. 16, 363–392. 10.1146/annurev.es.16.110185.002051

[B4] Bouchet A. S. Laperche A. Bissuel-Belaygue C. Snowdon R. Nesi N. Stahl A. (2016). Nitrogen use efficiency in rapeseed: A review. Agron. Sustain. Dev. 36, 1–20. 10.1007/s13593-016-0371-0

[B5] Chen G. Dresbøll D. B. Thorup-Kristensen K. (2021). Dual labelling by ^2^H and ^15^N revealed differences in uptake potential by deep roots of chicory. Rhizosphere. 19, 100368. 10.1016/j.rhisph.2021.100368

[B6] Dercon G. Clymans E. Diels J. Merckx R. Deckers J. (2006). Differential ^13^C isotopic discrimination in maize at varying water stress and at low to high nitrogen availability. Plant Soil. 282, 313–326. 10.1007/s11104-006-0001-8

[B7] Dresbøll D. B. Rasmussen I. S. Thorup-Kristensen K. (2016). The significance of litter loss and root growth on nitrogen efficiency in normal and semi-dwarf winter oilseed rape genotypes. F. Crop. Res. 186, 166–178. 10.1016/j.fcr.2015.12.003

[B8] Drew M. C. Saker L. R. Ashley T. W. (1973). Nutrient supply and the growth of the seminal root system in barley: I. The effect of nitrate concentration on the growth of axes and laterals. J. Exp. Bot. 24, 1189–1202. 10.1093/jxb/24.6.1189

[B9] Farquhar G. D. Ehleringer J. R. Hubick K. T. (1989). Carbon isotope discrimination and photosynthesis. Annu. Rev. Plant Physiol. Plant Mol. Biol. 40, 503–537. 10.1146/annurev.pp.40.060189.002443

[B10] Farquhar G. D. Richards R. A. (1984). Isotopic composition of plant carbon correlates with water-use efficiency of wheat genotypes. Aust. J. Plant Physiol. 11, 539–552. 10.1071/PP9840539

[B11] Garrigues E. Doussan C. Pierret A. (2006). Water uptake by plant roots: I - Formation and propagation of a water extraction front in mature root systems as evidenced by 2D light transmission imaging. Plant Soil 283, 83–98. 10.1007/s11104-004-7903-0

[B12] Gonzalez-Dugo V. Durand J.-L. Gastal F. (2010). Water deficit and nitrogen nutrition of crops. A review. Agron. Sustain. Dev. 30, 529–544. 10.1051/agro/2009059

[B13] Gorska A. Ye Q. Holbrook N. M. Zwieniecki M. A. (2008). Nitrate control of root hydraulic properties in plants: translating local information to whole plant response. Plant Physiol. 148, 1159–1167. 10.1104/pp.108.122499 18753287 PMC2556825

[B14] Griffiths M. Wang X. Dhakal K. Guo H. Seethepalli A. Kang Y.. (2022). Interactions among rooting traits for deep water and nitrogen uptake in upland and lowland ecotypes of switchgrass (*Panicum virgatum* L.). J. Exp. Bot. 73, 967–979. 10.1093/jxb/erab437 34604906 PMC8793874

[B15] Haberle J. Svoboda P. Krejčov,á J. (2006). Uptake of mineral nitrogen from subsoil by winter wheat. Plant Soil Environ. 52, 377–384. 10.17221/3455-pse32062254

[B16] Hashemian M. Ryu D. Crow W. T. Kustas W. P. (2015). Improving root-zone soil moisture estimations using dynamic root growth and crop phenology. Adv. Water Resour. 86, 170–183. 10.1016/j.advwatres.2015.10.001

[B17] Hodge A. Robinson D. Griffiths B. S. Fitter A. H. (1999). Why plants bother: Root proliferation results in increased nitrogen capture from an organic patch when two grasses compete. Plant, Cell Environ. 22, 811–820. 10.1046/j.1365-3040.1999.00454.x

[B18] Jensen L. S. Christensen L. Mueller T. Nielsen N. E. (1997). Turnover of residual 15N-labelled fertilizer N in soil following harvest of oilseed rape (*Brassica napus* L.). Plant Soil. 190, 193–202. 10.1023/A:1004253611044

[B19] Khan S. Anwar S. Kuai J. Ullah S. Fahad S. Zhou G. (2017). Optimization of nitrogen rate and planting density for improving yield, nitrogen use efficiency, and lodging resistance in oilseed rape. Front. Plant Sci. 8, 1–9. 10.3389/fpls.2017.00532 28536581 PMC5423294

[B20] Kirkegaard J. A. Lilley J. M. Berry P. M. Rondanini D. P. (2021). “Canola,” in Crop Physiology Case Histories for Major Crops, eds V. O. Sadras and D. F. Calderini (Academic Press), 518–549. 10.1016/B978-0-12-819194-1.00017-7

[B21] Kirkegaard J. A. Lilley J. M. Howe G. N. Graham J. M. (2007). Impact of subsoil water use on wheat yield. Aust. J. Agric. Res. 58, 303–315. 10.1071/AR06285

[B22] Kuhlmann H. Barraclough P. B. Weir A. H. (1989). Utilization of mineral nitrogen in the subsoil by winter wheat. Zeitschrift für Pflanzenernährung und Bodenkd. 152, 291–295. 10.1002/jpln.19891520305

[B23] Landl M. Schnepf A. Uteau D. Peth S. Athmann M. Kautz T.. (2019). Modeling the impact of biopores on root growth and root water uptake. Vadose Zo. J. 18, 1–20. 10.2136/vzj2018.11.0196

[B24] Li S. X. Wang Z. H. Malhi S. S. Li S. Q. Gao Y. J. Tian X. H. (2009). “Chapter 7 nutrient and water management effects on crop production, and nutrient and water use efficiency in dryland areas of China,” in Advances in Agronomy, Sparks, D. L. (eds). San Diego: Elsevier Academic Press Inc. p. 223–265. 10.1016/S0065-2113(09)01007-4

[B25] Li W. D. Hou J. L. Wang W. Q. Tang X. M. Liu C. L. Xing D. (2011). Effect of water deficit on biomass production and accumulation of secondary metabolites in roots of Glycyrrhiza uralensis. Russ. J. Plant Physiol. 58, 538–542. 10.1134/S1021443711030101 32214752 PMC7089503

[B26] Lilley J. M. Kirkegaard J. A. (2016). Farming system context drives the value of deep wheat roots in semi-arid environments. J. Exp. Bot. 67, 3665–3681. 10.1093/jxb/erw093 26976814 PMC4896360

[B27] Lynch J. Marschner P. Rengel Z. (2012). “Chapter 13 - Effect of Internal and External Factors on Root Growth and Development,” in Marschner's Mineral Nutrition of Higher Plants (Third Edition), Marschner, P. (eds). San Diego: Academic Press. p. 331–346. 10.1016/B978-0-12-384905-2.00013-3

[B28] Lynch J. P. Wojciechowski T. (2015). Opportunities and challenges in the subsoil: Pathways to deeper rooted crops. J. Exp. Bot. 66, 2199–2210. 10.1093/jxb/eru508 25582451 PMC4986715

[B29] Mueller N. D. Gerber J. S. Johnston M. Ray D. K. Ramankutty N. Foley J. A. (2012). Closing yield gaps through nutrient and water management. Nature. 490, 254–257. 10.1038/nature11420 22932270

[B30] Plett D. C. Ranathunge K. Melino V. J. Kuya N. Uga Y. Kronzucker H. J. (2020). The intersection of nitrogen nutrition and water use in plants: new paths toward improved crop productivity. J. Exp. Bot. 71, 4452–4468. 10.1093/jxb/eraa049 32026944 PMC7382376

[B31] Quemada M. Gabriel J. L. (2016). Approaches for increasing nitrogen and water use efficiency simultaneously. Glob. Food Sec. 9, 29–35. 10.1016/j.gfs.2016.05.004

[B32] Rasmussen C. R. Kulmatiski A. (2021). Improving inferences from hydrological isotope techniques. Trends Plant Sci. 26, 206–209. 10.1016/j.tplants.2020.12.013 33454210

[B33] Rasmussen C. R. Thorup-Kristensen K. Dresbøll D. B. (2020). Uptake of subsoil water below 2 m fails to alleviate drought response in deep-rooted Chicory (*Cichorium intybus* L.). Plant Soil. 446, 275–290. 10.1007/s11104-019-04349-7

[B34] Rasmussen I. S. Dresbøll D. B. Thorup-Kristensen K. (2015). Winter wheat cultivars and nitrogen (N) fertilization-Effects on root growth, N uptake efficiency and N use efficiency. Eur. J. Agron. 68, 38–49. 10.1016/j.eja.2015.04.003

[B35] Rathke G. W. Behrens T. Diepenbrock W. (2006). Integrated nitrogen management strategies to improve seed yield, oil content and nitrogen efficiency of winter oilseed rape (*Brassica napus* L.): A review. Agric. Ecosyst. Environ. 117, 80–108. 10.1016/j.agee.2006.04.006

[B36] R Core Team. (2019). R: A language and environment for statistical computing. R Foundation for Statistical Computing, Vienna, Austria. Available online at: https://www.R-project.org/

[B37] Riar A. Gill G. McDonald G. K. (2020). Rate of nitrogen rather than timing of application influence yield and NUE of canola in south australian mediterranean environments. Agronomy. 10, 7–9. 10.3390/agronomy10101505

[B38] Sadras V. O. Hayman P. T. Rodriguez D. Monjardino M. Bielich M. Unkovich M.. (2016). Interactions between water and nitrogen in Australian cropping systems: physiological, agronomic, economic, breeding and modelling perspectives. Crop Pasture Sci. 67, 1019–1053. 10.1071/CP16027

[B39] Schjoerring J. K. Bock J. G. H. Gammelvind L. Jensen C. R. Mogensen V. O. (1995). Nitrogen incorporation and remobilization in different shoot components of field-grown winter oilseed rape (*Brassica napus* L.) as affected by rate of nitrogen application and irrigation. Plant Soil. 177, 255–264. 10.1007/BF00010132

[B40] Sieling K. Kage H. (2010). Efficient N management using winter oilseed rape. A review. Agron. Sustain. Dev. 30, 271–279. 10.1051/agro/2009036

[B41] Sinclair T. R. Rufty T. W. (2012). Nitrogen and water resources commonly limit crop yield increases, not necessarily plant genetics. Glob. Food Sec. 1, 94–98. 10.1016/j.gfs.2012.07.001

[B42] Skinner R. H.. (2008). Yield, root growth, and soil water content in drought-stressed pasture mixtures containing chicory. Crop Sci. 48, 380–388. 10.2135/cropsci2007.04.0201

[B43] Smith A. G. Han E. Petersen J. Olsen N. A. F. Giese C. Athmann M.. (2020a). RootPainter: deep learning segmentation of biological images with corrective annotation. bioRxiv 2020.04.16.044461. 10.1101/2020.04.16.044461 PMC9804377 35851958

[B44] Smith A. G. Petersen J. Selvan R. Rasmussen C. R. (2020b). Segmentation of roots in soil with U-Net. Plant Methods 16, 1–15. 10.1186/s13007-020-0563-0 32055251 PMC7007677

[B45] Smith C. J. Wright G. C. Woodroofe M. R. Smith C. J. Woodroofe M. R. (1988). The effect of irrigation and nitrogen fertilizer on rapeseed (*Brassica napus*) production in South-Eastern Australia - II. Nitrogen accumulation and oil yield. Irrig. Sci. 9, 15–25. 10.1007/BF00292140

[B46] Svoboda P. Haberle J. (2006). The effect of nitrogen fertilization on root distribution of winter wheat. Plant Soil Environ. 52, 308–313. 10.17221/3446-pse30479909

[B47] Taylor A. J. Smith C. J. Wilson I. B. (1991). Effect of irrigation and nitrogen fertilizer on yield, oil content, nitrogen accumulation and water use of canola (*Brassica napus* L.). Fertil. Res. 29, 249–260. 10.1007/BF01052393

[B48] Thorup-Kristensen K. Halberg N. Nicolaisen M. Olesen J. E. Crews T. E. Hinsinger P.. (2020a). Digging deeper for agricultural resources, the value of deep rooting. Trends Plant Sci. 25, 406–417. 10.1016/j.tplants.2019.12.007 31964602

[B49] Thorup-Kristensen K. Halberg N. Nicolaisen M. H. Olesen J. E. Dresbøll D. B. (2020b). Exposing deep roots: a rhizobox laboratory. Trends Plant Sci. 25, 418–419. 10.1016/j.tplants.2019.12.006 31974066

[B50] Thorup-Kristensen K. Magid J. Jensen L. S. (2003). Catch crops and green manures as biological tools in nitrogen management in temperate zones. Adv. Agron. 79, 227–302. 10.1016/S0065-2113(02)79005-6

[B51] Vandoorne B. Beff L. Lutts S. Javaux M. (2012). Root water uptake dynamics of *Cichorium intybus* var. sativum under water-limited conditions. Vadose Zo. J. 11, vzj2012.0005 10.2136/vzj2012.0005

[B52] Wang M. Ding L. Gao L. Li Y. Shen Q. Guo S. (2016). The interactions of aquaporins and mineral nutrients in higher plants. Int. J. Mol. Sci. 17, 1–16. 10.3390/ijms17081229 27483251 PMC5000627

[B53] Waraich E. A. Ahmad R. Yaseen Ashraf M. Saifullah S. Ahmad M. (2011). Improving agricultural water use efficiency by nutrient management in crop plants. Acta Agric. Scand. Sect. B Soil Plant Sci. 61, 291–304. 10.1080/09064710.2010.491954

[B54] Wasson A. P. Richards R. A. Chatrath R. Misra S. C. Prasad S. V. S. S. Rebetzke G. J.. (2012). Traits and selection strategies to improve root systems and water uptake in water-limited wheat crops. J. Exp. Bot. 63, 3485–3498. 10.1093/jxb/ers111 22553286

[B55] Yousfi S. Serret M. D. Márquez A. J. Voltas J. Araus J. L. (2012). Combined use of δ^13^C, δ^18^O and δ^15^N tracks nitrogen metabolism and genotypic adaptation of durum wheat to salinity and water deficit. New Phytol. 194, 230–244. 10.1111/j.1469-8137.2011.04036.x 22300532

